# Phenotyping of *E. coli *serotypes associated to oedema disease

**DOI:** 10.1186/1751-0147-50-13

**Published:** 2008-06-02

**Authors:** Sigbrit Mattsson, Per Wallgren

**Affiliations:** 1 National Veterinary Institute, SVA 751 89 Uppsala, Sweden

## Abstract

**Background:**

Oedema disease is a severe disease, mainly affecting recently weaned pigs. It is caused by *E. coli *strains that express fimbriae F18 and produce verotoxin 2e, mainly belonging to serotype O138, O139 or O141. The aim of this study was to compare *E. coli *isolates within these serotypes with respect to diversity.

**Methods:**

Faecal *E. coli *strains belonging to serotypes O138, O139 and O141 isolated during the period 1994–1998 from Swedish pigs aged less than 12 weeks were compared using a biochemical fingerprinting system. Aiming to compare the results obtained over time, also strains isolated during 1964–67 and 1975–80 were included in the study. The study comprised 129, 263 and 95 isolates of *E. coli *serotype O138, O139 and O141, respectively.

**Results:**

Biochemical phenotypes (BPTs) were defined. At each sampling occasion each herd could only contribute with one isolate per BPT. Consequently, all but one of identical BPTs identified at a specific sampling occasion was omitted. The final number of isolates from 1994–98 that was compared included 64, 182 and 41 isolates of serotypes O138, O139 and O141, respectively. Within each serotype, the dominating BPT included over 65% of the compared isolates, demonstrating a large dominance of one BPT per serotype. These dominating BPTs were also demonstrated in the material from the 1960ies and the 1970ies. Still, the presence of other common BPTs (especially within serotype O138 and O139) demonstrated a certain variation within serotype. In a herd severely affected by oedema disease, *E. coli *serotype O139 was easily demonstrated in diseased pigs but only rarely in apparently healthy weaners

**Conclusion:**

The results obtained demonstrate the presence of dominating BPTs within the oedema disease inducing serotypes. A stability of these BPTs over time was observed, presumably at least partly due to a never-ending access to naïve pigs. Still, the presence of other common BPTs indicates a variation over time, which visualises the importance of monitoring for this. Such studies should focus on pigs affected by oedema disease, because oedema disease inducing strains of *E. coli *were only rarely demonstrated in healthy pigs in a herd affected by oedema disease.

## Background

Oedema disease is caused by *E. coli *strains that possess fimbrial adhesion factors enable to produce toxin [1]. Most of these strains belong to a few serotypes and the fimbriae is often F18 (previously named F107) and produce verotoxin 2e (VT2e), also called shiga-like toxin IIe [2,3]. The *E. coli *bacteria multiply in the digestive tracts and colonize the small intestine [4,5]. Names like "oedema disease," "bowel oedema" and "gut oedema" have been coined because oedema in the submucosa of the stomach and the mesocolon often are prominent features of the disease [6]. The toxin may cause vascular lesions in the intestine, in the sub cutis and in the brain, leading to oedema in affected organs and to neurological symptoms [3]. The production of VT2e is temperature dependent and most effective at 37°C, whereas the VT2e production is dramatically reduced at temperatures below 30°C or above 42°C [7].

Oedema disease is highly contagious and mainly affect piglets 5–14 days after weaning, although outbreaks in both younger and older pigs have been recorded [8,9]. Clinical signs generally include sudden deaths, occasionally preceded by incoordination and/or swollen eyelids [6]. The course is intensive, but usually decline within 4–5 days after a sudden onset in a group of pigs [10,11]. The morbidity varies, but in average 30–40% of the recently weaned pigs tends to be affected [6]. Also the mortality of the affected pigs varies considerably, from almost none to 70% [12].

A contributing reason to why oedema disease mainly affects pigs close to weaning probably is stress caused by simultaneously loosing access to the dam and the protective IgA present in her milk [13-15], combined with a sudden change from a milk based diet to a cereal diet. Together, these stressors severely affect the intestinal microflora during the first two weeks following weaning [16-19]. Other predisposing factors include intensive husbandry, feed composition, temperature alterations and presence of other infectious agents [4,5,20], as well as presence of intestinal F18-receptors [21].

Several strains of *E. coli *have been discussed as inducers of oedema disease, but from a historical point of view the disease have been associated to principally four strains of *E. coli *belonging to three different serotypes: O138:K81, O139:K12, O141:K85a,b:H4 and O141:K85a,c:H4 [22,23]. During clinical outbreaks of oedema disease, one of these *E. coli *strains can generally be demonstrated as pure or almost pure cultures in affected pigs [24]. However, bacterial numbers may have declined in more protracted cases and a negative bacteriological result therefore does not exclude the diagnose of oedema disease [1].

For diseases caused by a limited number of serotypes of *E. coli*, such as oedema disease, serotyping is a valuable diagnostic tool [25], and Sweden has a long tradition of serotyping *E. coli *collected from young pigs affected by intestinal disorders, including oedema disease [26,27]. Aiming to increase the information even furhter, all strains associated to oedema disease (O138, O139 and O141) collected during the period 1994–98 were phenotyped with a biochemical finger printing system [28] and comparisons were made within serotypes. To compare the phenotypic variation over time, also strains from 1964–67 and 1975–80 were included in the study.

## Methods

### Samples

Faecal samples collected from pigs with some kind of intestinal disorder have throughout decades been monitored in Sweden. The samples have been spread on horse blood agar and bromine-cresol-purple agar. After incubation at 37°C for 18h, at least two colonies were cultivated in a beef extract broth (NVI product 311060) at 37°C for another 18h. The broth culture was then heated in 100°C for 1 hour and agglutinated with specific antisera from a panel of different *E. coli *serotypes [26]. This panel of serotypes is continuously updated in order to represent the most common pathogenic strains of *E. coli *in Sweden [29,27].

All isolates of *E. coli *serotypes O138, O139 and O141 from pigs younger than 12 weeks during 1994–98 were selected for further investigations (n = 487). For historical comparisons, also strains from 1964–67 and 1975–80 were scrutinised. (Table 1).

**Table 1:** The initial number of *E. coli *isolates committed for biochemichal fingerprinting during 1994 to 1998, and the final numbers included in these comparisons after excluding identical BPTs collected in herds at specific sampling occasions.

	*E. coli *serotype		
	
PhP-typings (n)	O138	O139	O141
***Samples collected 1994–98***			
Initial number	108	234	49
Excluded as duplicates	44	52	8
Final number	64	182	41
Number of common BPTs	5	8	2
			
***Historical references***			
Collected 1964–67	18	14	27
Collected 1975–80	3	15	19

The table also shows the number of common BPTs obtained within serotype

### Biochemical fingerprinting

The selected strains were phenotyped using a biochemical fingerprinting method. These tests were performed in microtiter plates containing 11 different dehydrated reagents (The Phene Plate System: PhP-system, Biosys AB, Stockholm, Sweden) based on the evaluation of the kinetics of biochemical reactions [28,30,31]. The reagents (Cellobiose, Lactose, Rhamnose, Deoxyribose, Sucrose, Sorbose, Tagatose, L-Arabitol, Melbionat, Gal-Lac and Ornithine) were chosen because they give a high discrimination between different BPTs of *E. coli*. The indicator in the substrate added to all wells was bromthymol blue (0.1% proteose peptone and 0.011% bromthymol blue in destilled water). The substrate solution turns yellow at acidic pH, green at neutral pH and blue at alkaline pH.

The 487 selected frozen strains were again cultured and serotyped. The biochemical fingerprinting was performed in microtiter plates and each plate analysed 8 isolates of *E. coli*. From each strain a single colony was inoculated to 375 μl substrate to a well in the first row of a microtiter plate. After at least 1 hour the contents in the wells were homogenised by mixing three times using a multi channel pipette with sterile tips and thereafter 25 μl was transferred to each of the other eleven wells in the row that contained the 11 reagents described above. The microtiter plates were covered with sterile lids and incubated at 37°C. The colour reaction in the plates was measured at 650 nm using a photometer (Titertek Multiscan MCC/340, Labsystems OY, Helsinki, Finland) after 16, 48 and 64 hours of incubation. The scores from all readings were added together and compared in a computer program [28].

### Definition of biochemical phenotypes (BPTs)

Isolates showing a similarity of at least 97.5% were considered to belong to the same biochemical phenotype (BPT). Isolates from the same herd and sampling occasion belonging to the same BPT were defined as duplicates and all but one of them were excluded before comparisons between herds and sampling occasions were made. Thus, each herd could only contribute with one isolate per BPT and sampling occasion.

BPTs identified at only one or two occasions were defined as single or double BPTs, respectively. BPTs identified at three or more occasions were defined as common BPTs.

### Comparisons of isolates of *E. coli *within herds over time

Whenever a specific serotype of *E. coli *was demonstrated at different time points from individual herds during 1994–98, the BPTs of the different occasions were compared with each other.

### Comparisons of isolates of *E. coli *originating from different decades

The strains originating from 1964–67 and 1975–80 were defined using the PhP-system, and the BPTs obtained were compared with the BPTs defined in the material from 1994–98.

### Additional samplings in a herd affected by oedema disease

Feacal samples were collected from a herd seriously affected with oedema disease where *E. coli *O139 previously had been demonstrated in 4 out of 5 diseased pigs at necropsy. During a period of six months, faecal samples were collected from 35 apparently healthy weaners aged nine weeks. *E. coli *were serotyped, and isolates belonging to O138, O139 or O141 were compared with the overall material by biochemical fingerprinting.

## Results

### Biochemical fingerprinting

As mentioned in material and methods, each herd could only contribute with one isolate per BPT and sampling occasion when comparisons between herds were made. Strains from the same herd belonging to the same BPT were defined as duplicates and all but one of them excluded before further comparisons were made (Table 1). Duplicate BPTs comprised 41% of the O138-isolates, 22% of the O139-isolates and 16% of the O141-isolates. After this exclusion the survey comprised 287 isolates from 1994–98 (O138, n = 64; O139, n = 182 and; O141, n = 41).

**Figure 1:**
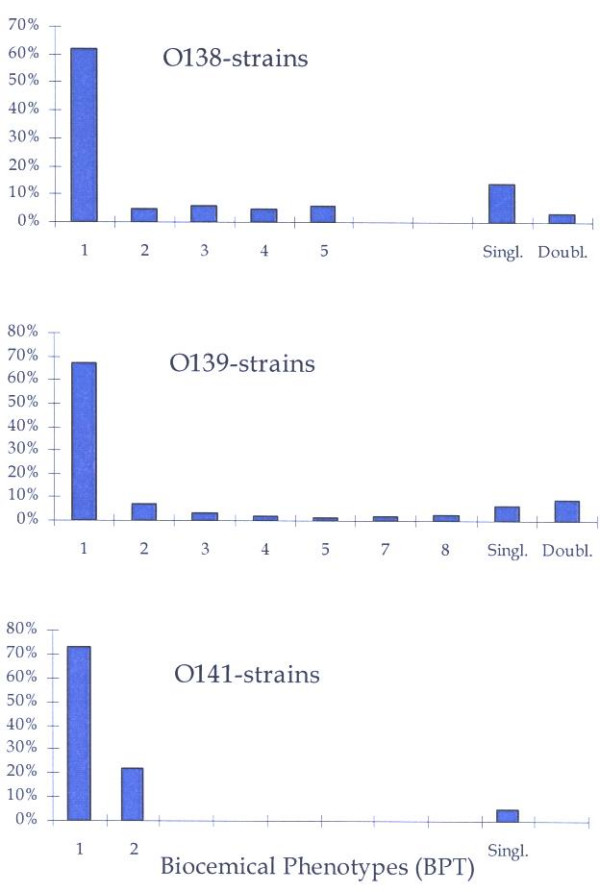
The distribution (%) of different biochemical phenotypes (BPTs) in *E coli *serotypes associated to oedema disease isolated in Sweden during 1994–1998. In each figure the numbers represent major BPTs, *i.e *BPTs diagnosed at more than two occasions. BPTs denoted singles and doubles represent BPTs demonstrated at one or two occasions in total.

When the final selection of strains collected during 1994–98 was examined with respect to phenotypic diversity, one dominating BPT was found within each serotype (Figure 1). Each dominating BPT comprised over 65% of the isolates within serotype (41 out of 64 O138-isolates = 66%; 120 out of 182 O139-isolates = 66%; and 30 out of 41 O141-isolates = 73%). All these dominant BPTs were also represented in the historical isolates collected during 1964–67 and 1975–80.

Apart from the dominant BPTs, 4 and 7 common BPTs were found within serotypes O138 and O139, respectively (Table 1 and Figure 1). None of these BPTs included more than 10% of the total number of isolates. There were also some BPTs that only were recorded occasionally. The latter category (isolated once or twice, *i.e*. singles or doubles) included around 15% of all isolates within these serotypes.

The phenotypic difference was less obvious within serotype O141. Only two BPTs included more than one isolate. Together these two phenotypes included 39 of the investigated 41 isolates (95%). The dominating BPT comprised 73% of the isolates and the second BPT 22%. The remaining 5% of the isolates were defined as single BPTs (Figure 1).

**Figure 2:**
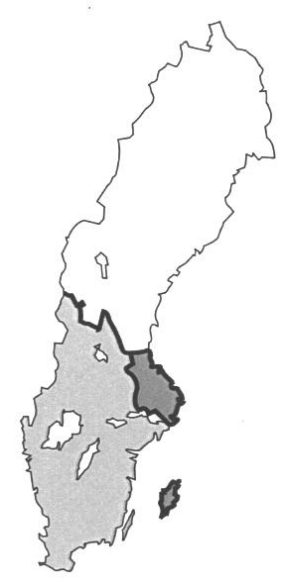
All oedema disease inducing strains of *E. coli *were collected in the shadowed part of Sweden. This area house 97% of the pigs in the country. The darker island indicates the island of Gotland. The darker area of the mainland indicates the county of Uppland.

The investigated *E. coli *isolates originated from the geographical regions Götaland and Svealand (Figure 2; shadowed area), which harbours 97% of the Swedish pig population. The dominating BPTs of O139 and O141 were both evenly spread over this area, including the island of Gotland (Figure 2, darker island). In contrast, the dominant BPT of O138 was only found in the southern and western parts of Sweden. The eastern part of the country had local minor BPTs of O138, the BPT denoted 2 was only found in Uppland (Figure 2, darker area of the mainland; n = 3, different herds) and the BPT denoted 4 was only demonstrated on the island of Gotland (n = 3, different herds).

### Relationships between isolates of *E. coli *within herds over time

During 1994–98, oedema disease inducing strains of *E. coli *was demonstrated at more than one occasion in 23 herds (Table 2). With respect to serotype O138 (n = 7), specific BPTs were repeatedly found in each herd. In five of them it was the national dominating BPT, and in one of these five herds two different common BPTs were repeatedly demonstrated. Regarding serotype O139, the dominating BPT was constantly diagnosed in 13 out of 15 herds. Serotype O141 was only diagnosed at two different occasions in one herd. At both occasions the dominating BPT was identified (Table 2).

**Table 2:** Comparions between *E. coli *strains related to oedema disease and repeatedly demonstrated within farms during 1994 – 1998.

			**Common**	**BPT**	**Clone**	
		
**Herd**	**Occasions when isolated**	**1994**	**1995**	**1996**	**1997**	**1998**
***O138***						
O138–1	4	-	-	1 & 3, 3	1, 1	-
O138–2	2	-	-	1	-	1
O138–3	3	-	-	-	1	1, 1
O138–4	2	-	-	-	1	1
O138–5	2	-	-	-	-	1, 1
O138–6	2	-	2	-	-	2
O138–7	4	-	-	-	5	5, 5, 5
						
***O139***						
O139–1	3	1, 1, 1	-	-	-	-
O139–2	2	1	1	-	-	-
O139–3	2	1	-	1	-	-
O139–4	3	1, 1	-	-	1	-
O139–5	2	1	-	-	-	1
O139–6	2	-	1, 1	-	-	-
O139–7	3	-	1, 1	1	-	-
O139–8	3	-	-	1, 1, 1	-	-
O139–9	3	-	-	1, 1	-	1
O139–10	4	-	-	1	1, 1, 1	-
O139–11	3	-	-	1, 1	1 & 5	-
O139–12	2	-	-	1	1	-
O139–13	2	-	-	-	-	1, 1
O139–14	2	2, 8	-	-	-	-
O139–15	2	-	-	-	Single	2
						
***O141***						
O141–1	2	-	-	-	1	1

Different samplings within years are separated by, and when two BPTs were recorded at the same occasion they are merged with & Within serotype, each digit denotes a certain common BPT of which the digit 1 represents the national dominant BPT of the period

### Relationships between isolates of *E. coli *originating from different decades

In total, 79 out of the 96 biochemically fingerprinted *E. coli *strains from the 1960ies and the 1970ies (83%) belonged to BPTs that also were demonstrated in the 1990ies (Table 3). As seen in that table, a majority of the BPTs defined within serotype O141 during the early years was equivalent to the dominating BPT of 1994–98. During the 1960ies, 89 % of the isolates within serotype O138 were either identical to the dominating BPT (33%) or matched other common PBTs (56%) of the 1990ies. With respect to serotype O139, 10% of the isolates from the early years were identical to the dominating BPT of the 1990ies and another 62% matched other common BPTs of that time. However, 28% (8 out of 29) of the early isolates belonged to BPTs not identified in the 1990ies (Table 3).

**Table 3:** Similarity between *E. coli *strains related to oedema disease over time.

Strains collected 1994–98;	***O138***		***O139***		***O141***	
*Number of common BPTs*	5		8		2	
*BPTs with > 5% of all isolates*	5		2		2	
Match to BPTs from 94–98	n	%	n	%	n	%
***Isolates collected 1964–67***	***18***	***100***	***14***	***100***	***27***	***100***
Dominating BPT 94–98	6	33	1	7	22	81
Second largest BPT 94–98	0	0	1	7	1	4
Other PBTs with > 5% 94–98	9	50	-	-	-	-
Other BPTs identified 94–98	1	6	10	71	1	4
BPTs not identified 94–98	2	11	2	14	3	11
						
***Isolates collected 1975–80***	***3***	***100***	***15***	***100***	***19***	***100***
Dominating BPT 94–98	0	0	2	13	9	47
Second largest BPT 94–98	0	0	0	0	1	5
Other PBTs with > 5% 94–98	0	0	-	-	-	-
Other BPTs identified 94–98	1	33	7	48	7	37
BPTs not identified 94–98	2	67	6	40	2	11

Biochemical phenotypes collected during the 1960ies and 1970ies compared to BPTs collected during the 1990ies

### Additional samplings from the herd affected by oedema disease

The *E. coli *O139 strain demonstrated in four out of the five pigs diagnosed for oedema disease at necropsy belonged to the national dominating BPT. In contrast, *E. coli *serotype O139 was only demonstrated in 2 out of the 35 investigated healthy weaners (Table 4). Both these isolates belonged to the national dominating BPT and were collected from surviving pigs in one litter previously affected by oedema disease.

**Table 4:** Serotypes of *E. coli *demonstrated in faecal samples collected from apparently healthy weaners in a herd severely affected by oedema disease caused by *E. coli *serotype O139.

Serotype	O139	O8	O98	O149	O157	O?
***Samples***						
35	2	3	4	4	1	21
(%)	5.7%	8.6%	11.4%	11.4%	2.9%	60.0%

The two isolates of *E. coli *O139 were obtained from two survivors in a litter affected by oedema disease. These isolates belonged to the same BPT that previously had been demonstrated in pigs from the herd affected by oedema disease.  The diagnostic panel included 18 of the most common pathogenic serotypes in the country

## Discussion

Biochemical fingerprinting has frequently been used to measure the diversity of *E. coli *or coliforms in the gastrointestinal tracts of individual pigs at stressful situations such as weaning or allocation [17,18,32]. However, the method has also been successfully used when relationships between different isolates of specific bacteria have been investigated [33,34] and it has been used to update diagnostic panels for relevant serotypes in the national monitoring program for *E. coli *[29,27].

The present study showed that one specific BPT of oedema disease inducing *E. coli *generally was present within herds at each occasion. Further, a survival of that BPT was demonstrated when strains collected from individual herds were compared over time. These results coincide with reports indicating that a single serotype [24] or a single clone [25,26] is the inducer of oedema disease at herd level. Further, the presence of national dominant BPTs was observed, and at least 65% of the isolates within a serotype belonged to that BPT. As these BPTs were present in the country already during the 1960ies, a national stability within serotype was proven over time. One reason for this probably is that these BPTs are well adapted for survival. Another important factor probably is that these dominating BPTs also continuously have had access to naïve piglets that not yet have developed immunity towards these BPTs, which in turn will reduce the selective pressure.

This continuous dominance of specific BPTs over time was especially evident within serotype O141. Despite that the dominating BPTs of the 1990ies within serotypes O138 and O139 also were demonstrated frequently in the historical samples, a variation of dominating BPTs over time was actually indicated. Still, a majority of the historical BPTs were also present in 1994–98, indicating that these BPTs are well adapted for survival. However, the presence of several minor BPTs indicated an occurrence of genetic drift. This was especially evident within serotype O138 where the dominant BPT was restricted to the south-western part of Sweden, whereas unique BPTs were found both in Uppland and at the island of Gotland.

The presence of several common but minor BPTs calls for a continuous monitoring of the oedema disease inducing strains of *E. coli *in order to follow their evolution. Serotyping is also important since production of verotoxin has been reported also for *E. coli *isolates of serotypes rarely associated to oedema disease, such as O2, O120, O121, O149 and O157 [35,36]. The importance of serotyping has recently been illustrated in the province of Guizhou in China where the most common serotype diagnosed at oedema disease was *E. coli *serotype O82 [37]. Further, 42% of the strains collected from outbreaks of oedema disease in Iowa in the United States between 1996 and 2000 belonged to serotype O147 [38].

However, the serotypes classically correlated to oedema disease should still be focused. The most common serotype in Danish pig herds during the years 1988–1995 was serotype O139 originating from one clone [39], followed by a few herds infected with serotype O138 and O141 [40]. In another report from China, 8 out of 11 *E. coli *strains cultivated from pigs with oedema disease belonged to serotype O139 [41].

As evident from this study, serotype O139 is still the most frequently demonstrated oedema disease inducing serotype also in Sweden. Together with the presence of dominating BPTs this unfortunately indicate problems to trace the origin of the infection when new herds are affected by oedema disease. Not least since our results indicate that it must be taken into account that oedema disease inducing strains also may be present in apparently healthy herds [1]. The faeces of weaners contain around 10^5 ^*E. coli *per gram [42,18]. Healthy pigs may therefore harbour low numbers of oedema disease inducing strains of *E. coli *that almost certainly will escape detection in their highly varied intestinal flora even when looked for. Indeed *E. coli *O139 was only demonstrated in two out of 35 apparently healthy weaners in a herd severely affected by oedema disease, despite that this strain easily was demonstrated in diseased pigs of the herd. Interestingly, both these healthy pigs were survivors in a litter affected by oedema disease.

## Conclusion

To conclude, the BPTs of *E. coli *that induce oedema disease appear to be rather few and also quite stable. Still a number of other BPTs are continuously present, which indicate a variation over time and calls for monitoring. Further, serotyping of *E. coli *strains isolated from pigs with oedema disease is still a valuable tool since new serotypes recently have been linked to the disease.

## Authors' contributions

SM carried out the analysis performed and was the first author, PW made the study design in collaboration with SM and was the senior scientist. Both authors have contributed significantly to the work
